# Femoral hernia in the era of TAVI – a potential obstacle for transfemoral approach: a case report and literature review

**DOI:** 10.1186/s12893-020-0693-3

**Published:** 2020-02-10

**Authors:** Piotr Marciniuk, Dariusz Jagielak, Jan Rogowski, Piotr Gumiela, Kamil Bury

**Affiliations:** Department of Cardiac and Vascular Surgery, Medical University of Gdańsk, Dębinki 7, 80-952 Gdańsk, Poland

**Keywords:** Groin hernia, Femoral hernia, TAVI, Endovascular, Puncture

## Abstract

**Background:**

Transcatheter aortic valve implantation (TAVI) via total percutaneous transfemoral approach is an increasingly common technique for aortic stenosis treatment. It is primarily indicated in elderly with serious comorbidities. The epidemiology of these patients tends to overlap with the incidence of femoral hernia (FH). The appearance of hernia sac at the approach site and insufficient preoperational examination can lead to serious complications. We present the first-ever reported case of subsequent femoral hernia repair during transfemoral TAVI.

**Case presentation:**

This report presents a case of FH/TAVI coincidence and literature review of its epidemiology. Literature review was performed to analyze similarities of femoral hernia and TAVI. The case describes an 84-year old female referred for elective TAVI. Intraoperation incarcerated femoral hernia was noticed and directly repaired. Further TAVI steps were performed on regular basis. A 2-year follow-up reported no local and general complications related to procedures.

**Conclusions:**

Unsuspected femoral hernia found subsequently with transfemoral TAVI may become a growing problem. The number of TAVI performed rises with indications expansion. Femoral hernia repairs constitute from 2 to 4% of all groin hernia. Both TAVI and FH are connected with elderly. Despite the fact of low FH incidence, growing number of TAVI performed and ageing of population, corresponds with higher possibility of complications. Most of these complications may end up fatal as they would involve high-risk patients. Insufficient attention is paid by cardiologists to the possible hernia appearance in the access site as this issue has been hardly ever presented in literature. Concomitant FH in TAVI patients should always be excluded in order to avoid serious complications. The case we report presents a successful subsequent FH repair during TAVI procedure. Further studies have to be conducted to provide data on how such problems ought to be managed.

## Background

Since the first transcatheter aortic valve implantation (TAVI) being performed by Alain Cribier and colleagues in 2002 [1], the technique has gained global support in over 40 countries with more than 200,000 implantations performed [2–4]. The rapid development of this technology has been triggered by the need for less invasive treatment in patients excluded from conventional open surgical valve replacement due to high perioperative risk. Nowadays TAVI is the standard of care for high risk or inoperable patients and is a valid alternative to surgery for selected high-risk but operable patients with symptomatic aortic stenosis [5–7]. With growing experience, a shift towards the treatment of younger patients with fewer comorbidities and lower surgical risk scores is observed [8, 9]. Femoral artery access for transfemoral TAVI is achieved either by surgical approach (arterial cutdown) or through a totally percutaneous approach (arterial puncture and closure with vascular closure devices) [10]. Worldwide tendency to miniaturization of surgical techniques causes the percutaneous approach to be more willingly chosen by surgeons and accepted by patients.

The incidence of femoral hernia in relation to all groin hernias is reported to be 2 to 4% in adult population [11–17]. They are far more common in females than males with a ratio of around 2:1. Epidemiological studies show a great increase of its incidence with age in both genders [18–20]. Femoral hernia sac protrudes through the femoral canal projecting in the femoral triangle nearby common femoral artery. This artery is commonly used as an approach in transfemoral TAVI. Thus, performing percutaneous TAVI at this localization in a patient with insufficient access site examination or occult hernia may lead to serious complications. Unaware surgeon can puncture the hernia sac which may cause gastrointestinal tract perforation, hemorrhage, peritonitis or even death. Seyfarth et al. presented a case of such scenario during percutaneous transluminal angioplasty through the groin [21]. This was the first and only report of such possible complication ever described. The femoral hernia sack in this case contained incarcerated small bowel loop. The contents have been punctured while achieving antegrade femoral artery access leading to peritonitis and urgent laparotomy. Final management resulted in partial small bowel resection.

Complex literature review has been done to study characteristics of femoral hernia and TAVI procedures in population. Epidemiological similarities were found. However, no data have ever been published on this concomitance and surgeons cannot base their decisions on any evidence. The study, therefore, aims to highlight the possible problem of femoral hernia incidence in patients having TAVI performed. We report one case of FH and TAVI coexistence and present literature review.

## Case presentation

**Fig. 1:**
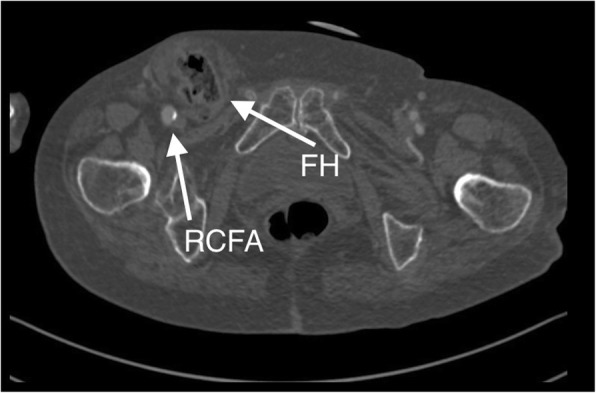
Computed tomography angiography (CTA) transverse cross-sectional image. Right common femoral artery (RCFA), femoral hernia (FH) sack

An 84-year old Caucasian female was admitted to the Department of Cardiac and Vascular Surgery due to elective TAVI for severe aortic stenosis treatment. The patient had surgical aortic valve replacement declined by cardiac surgery team because of hemodynamic instability and comorbidities (hypertension, narrow complex tachycardia, previous myocardial infarction). The incidence of FH history was well known by the team. Patient presented minor bulge in the right groin which was easily manually reduced. No previous history of strangulation or incarceration has been reported. Computed tomography angiography (CTA) scan was performed 5 months before the procedure in order to plan valve repair. A femoral hernia of 50x53mm in size in the right groin was confirmed (Fig. 1). The sack contained small bowel loops with proper contrast perfusion with no signs of strangulation. Hernia ring of 30 mm was measured. Due to lack of symptoms, easy manual reduction and patient’s preference, no elective hernia repair was planned.

**Fig. 2:**
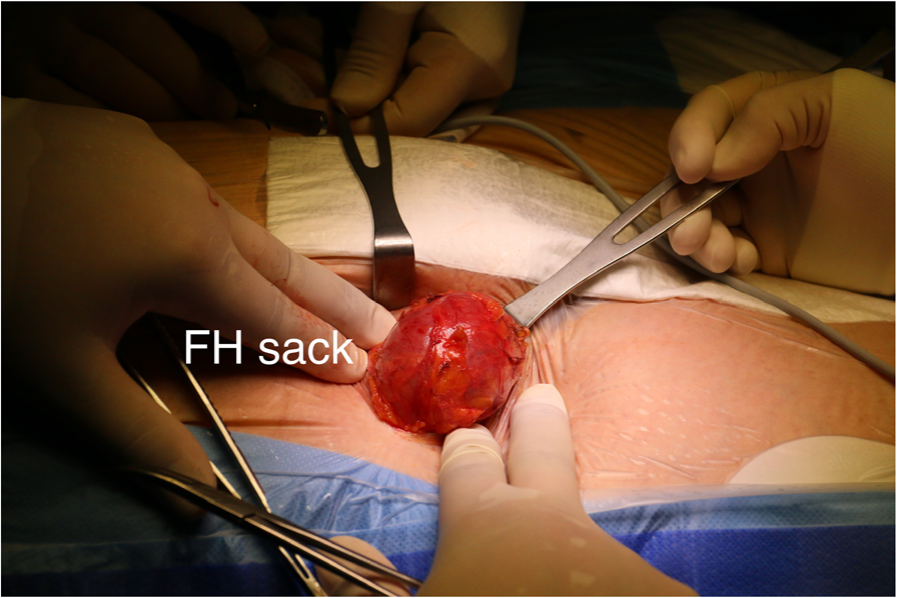
Femoral hernia sack protrusion after right groin cut-down

**Fig. 3:**
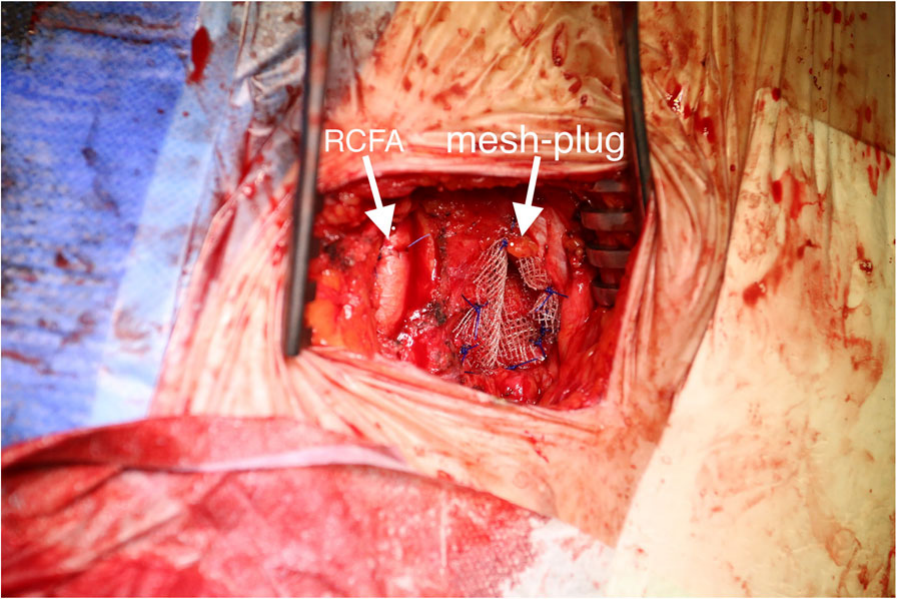
Surgical site image after complete repair. Hernial sack reinforced with a mesh-plug. Right common femoral artery (RCFA) puncture closed by direct vascular suture

TAVI procedure was scheduled and ordinarily performed. Patient has been fully sedated with intubation by the anesthesiology team. Through standard transverse left groin cutdown diagnostic devices were placed into left femoral vein and left common femoral artery without any complications. Surgical team noticed a significant bulge in the right groin. Physical examination evinced an incarcerated femoral hernia with no response to manual reduction. An intraoperation general surgery consultation has been called. No success in hernia reduction was achieved again. A suspicion of incarceration occurring during sedation induction has been drawn. Consulting general surgeon indicated a FH repair prior further TAVI steps. Right transverse cutdown was performed revealing a tense hernial sac of 100x70mm in size which again could not be reduced manually. Hernial sac was carefully dissected, right common femoral artery and vein dissected. No signs of strangulation have been observed. The sac was resected, its contents reduced into peritoneal cavity (Fig. 2). Hernial ring was closed and reinforced using Rutkow-Robbins mesh-plug technique (Fig. 3). Following TAVI steps were performed with no complications and valve successfully replaced. No post operation and 2-year follow-up local and general complications were reported.

## Discussion and conclusion

Majority of studies rely on overall groin hernias repair databases comprising of inguinal hernias mostly. Thus, the real femoral hernia incidence is likely to be lower than 2 to 4% since this estimate is unsettled by the high percentage of surgically-treated femoral hernias compared to inguinal hernias [22]. The low incidence of femoral hernias causes them difficult to study in randomized trials and existing literature is mainly based on patient series of varying sizes. Nevertheless, femoral hernia incidence is low, the amount of TAVI performed increases. Smith et al. proved its effectiveness in high-risk older patients [5]. Furthermore, indications are still being expanded covering lower-risk patients. The old age of both groups of patients speak for suspected increasing concomitance of TAVI and FH. The large national study conducted by Burcharth et al. reports the greatest FH repair prevalence in females aged 80–90 which is also a typical age of TAVI patients commonly confirmed by entire reviewed literature [18]. Another issue is that the deployment of the main system during TAVI (the valve itself) is predominantly performed though the right groin. Unfortunately femoral hernia occurs more often on the same side [13]. The opposite groin is used as well for diagnostic catheters access. As both groin access is essential to perform TAVI procedure, a potential FH on any side can be punctured leading to serious complications. Thus, there is no way to decrease hernia encounter possibility regarding its side incidence preference.

Femoral hernias are repaired more often than inguinal hernias since the risks of serious and potentially lethal complications such as strangulation and bowel resection are unacceptably high [23]. Dahlstrand et al. proved that femoral hernia repair should be performed as an elective surgery [23]. Of course, an emergency surgery is considered as a result of strangulation or incarceration. However, an iatrogenic perforation of the hernial sac with an access device during TAVI procedure should also be considered. Femoral hernia sac can contain pre peritoneal fat and abdominal or pelvic mass (e.g. intestines) [24]. Puncture may lead to gastrointestinal tract perforation, hemorrhage and peritonitis [21]. Thus, an injury to the hernial sac may also require an emergency repair [21]. Even though the incidence of femoral hernia is rather low, cardiac surgeons have to stay aware of this possibility. Adding a hernia sac complication to a high-risk patient having TAVI performed may become fatal. Evidence show a favorable effect of elective femoral hernia repairs to emergency ones. Gunnarsson et al. reported that elective surgery in older patients is a low operative risk choice regardless of age and sex [25]. Hence, we assume that a planned elective femoral hernia repair should be performed in patients with hernia diagnosed at the surgical table after performing groin cut section for transfemoral TAVI. Our experience shows an example of such management with a satisfactory outcome (see Case presentation). Of course, further studies should be performed in order to prove it.

A disturbing fact is that a femoral hernia can be missed on anamnesis, physical examination and imaging of the transfemoral TAVI approach site. Most patients with reducible FH complain of pain, discomfort in the groin or can stay asymptomatic. On physical examination it exhibits as a smooth lump on the medial side of the thigh beneath the inguinal ligament [24]. There can be some explanations for missed femoral hernias. Patients being admitted to have TAVI performed are not sufficiently interviewed in terms of discomfort or pain in the groin. Cardiac surgeons can be unaware of such issue, because literature have never embraced this topic. What is more femoral hernias are usually difficult to diagnose on physical examination [26]. It can mean an occult femoral hernia (a nonpalpable hernia) or lack of experience in hernia examination. Hair et al. concluded that practitioners who do not deal with hernia patients are rather inefficient in diagnosis especially when an elective not urgent strangulated hernia is considered [27]. Moreover, differentiation between FH and IH is also an issue. In this case a cooperation with a hernia or general surgeon may be a solution.

Femoral hernias have a great strangulation percentage comparing to IH [14]. Thus an urgent repair after diagnosis is recommended in contrast to inguinal hernias and watchful waiting [23, 28]. However, there is no prospective studies regarding the natural history of an asymptomatic FH. This is an issue because an oversight of a femoral hernia in a possible complication-free TAVI can conceal following strangulation symptoms. The pain in the groin may be associated with the TAVI system insertion. Hence, timing of asymptomatic FH repair in a patient with a planned TAVI turns to be a challenging and multidisciplinary problem. Every patient has a pre TAVI CTA performed to evaluate vascular status, potential anatomic considerations and best surgical approach. There is ambivalent evidence on CTA usefulness in groin hernias diagnosis with European Hernia Society guidelines specifying a non-significant role of such imaging [28]. Thus, a preoperational ultrasonography as an assessment of the approach sites occur to be a satisfactory solution. Its positive predictive value was proved by Robinson et al. [29]. The advantage is that it can be performed directly before obtaining access to ensure a safe approach. Combining these two methods gives the best chance to avoid possible access related complications.

The type of transfemoral TAVI approach has been compared by Bernardi et al. and showed no significant differences in outcomes [10]. Percutaneous access is usually performed by more experienced operators. It obviously reduces invasiveness and is more willingly accepted by patients. On the other hand, it is considered as less controlled resulting in impaired safety. If performed in a patient with unsuspected femoral hernia, the endovascular systems may pierce through the hernia while aiming artery or vein. This fact may be overlooked as the patient is sedated and with local analgesia administered. Eventually the artery is closed with a vascular closure device. No inspection is done into the access site which may have fatal outcomes as the symptoms of the hernia sac perforation would appear later resulting in an emergency revision [21]. Such scenario undermines the equality of percutaneous and open approach. Our case reports a promising aspect of groin cutdown approach. It allowed to visualize the hernia sac which resulted in swift hernia repair. However further studies have to be conducted to deliver proper evidence.

Although no data on the field of FH and TAVI concomitance have been reported before, we assume that this problem may grow in number. Except from TAVI a similar obstacle can occur in other endovascular procedures with femoral approach such as EVAR or any other percutaneous transfemoral angioplasty.

Concomitant femoral hernia in patients being treated with transfemoral TAVI due to severe aortic stenosis treatment should always be excluded before the procedure. There are similarities in patient’s demographics. No data have ever been published regarding this topic. Hence further prospective and observational studies have to be conducted in order to investigate concomitance of FH and TAVI or any other transfemoral vascular procedure and to elaborate proper management protocol. We suggest a broad multidisciplinary approach before acting. Involvement of cardiac surgeon and general surgeon with hernia repair experience should be advocated in patients’ examination. Moreover, imaging of the femoral access site must not overlook hernia assessment.

## Data Availability

The data that support the findings of this study are available from University Clinical Centre, Gdansk but restrictions apply to the availability of these data, which were used under license for the current study, and so are not publicly available. Data are however available from the authors upon reasonable request and with permission of University Clinical Centre, Gdansk.

## Abbreviations

CTA: Computed tomography angiography
EVAR: Endovascular aneurysm repair
FH: Femoral hernia
IH: Inguinal hernia
RCFA: Right common femoral artery
TAVI: Transcatheter aortic valve implantation
